# Supplementary material for: Parameter estimation for nonlinear sandwich system using instantaneous performance principle
Source: PLoS One. 2022 Dec 14;17(12):e0271160. doi: 10.1371/journal.pone.0271160 (PMC9749997; doi:10.1371/journal.pone.0271160)
Supplement: Attachment — Submitted filename: PONE-D-22-08046-suggestions.pdf [file pone.0271160.s002.pdf]

Suggestions

In this work, an instantaneous performance scheme of parameter estimation is introduced for nonlinear sandwich system. To achieve the above purpose, the estimation error information reflecting the transient performance of parameter estimation is procured using the developed some intermediate variables. Then, a predefined constraint function is used to prescribe the error convergence boundary, in which the convergence rate is lifted. The advantages and usefulness of the article are proved by examples.

The major novelty of this paper is to use preset performance technology. As far as I am concerned, the identification algorithm pays most attention to the steady-state performance, and it is difficult to study the transient performance.

(1) In Assumption 1, the author provides some assumptions, but does not offer the role of these assumptions.

(2) The author proposes the estimation algorithm containing some parameters, and some parameter selection criteria can be added, so as to increase the integrity of the original manuscript.

(3) As we all know, the forgetting factor can increase data utilization. Please clarify the advantages of variable fading factor in this article.

(4) The filter v is chosen upon the cutoff frequency. In engineering application, 100 Hz is selected in general. Then, 50 Hz is chosen as cutoff frequency. Is it better to use v based on the sampling frequency?

(5) In Experiment section, the identification model for servo system should be added to increase the readability of the study.
