# Supplementary material for: Parameter estimation for nonlinear sandwich system using instantaneous performance principle
Source: PLoS One. 2022 Dec 14;17(12):e0271160. doi: 10.1371/journal.pone.0271160 (PMC9749997; doi:10.1371/journal.pone.0271160)
Supplement: Attachment — Submitted filename: Comment.docx [file pone.0271160.s003.docx]

Comment

The almost existing identification algorithms do not consider the prescribed error bound on the parameter estimation error information, which may result in the poor transient performance of parameter estimation during the identification process. In this paper, a novel identification scheme is presented for nonlinear sandwich model, which is implemented by using prescribed performance function and error transformation technique. The numerical example and experiment results validate that the proposed scheme can provide more accurate parameter estimation and better transient performance than the existing methods.

Comment 1: If the noise attribute is unknown, how to design the de-noising filter of this paper.

Comment 2: There exist two linear subsystems in this paper, the assumptions on the two linear systems should be stated as formal assumptions.

Comment 3: In Eq.(15), please remove the necessary derivation steps and give the results directly, because these formulas are easy to deduce.

Comment 4: The presentation and language need be improved.

Comment 5: The auxiliary model method can indeed solve the problems in this paper, but the specific solution block diagram needs to be provided.

Comment 6: In the process of proving the convergence of the proposed algorithm, I am interested in the meaning of the symbolic representation of “O” in Eq.(31).
